# Multi-Targeted Antiangiogenic Tyrosine Kinase Inhibitors in Advanced Non-Small Cell Lung Cancer: Meta-Analyses of 20 Randomized Controlled Trials and Subgroup Analyses

**DOI:** 10.1371/journal.pone.0109757

**Published:** 2014-10-16

**Authors:** Wenhua Liang, Xuan Wu, Shaodong Hong, Yaxiong Zhang, Shiyang Kang, Wenfeng Fang, Tao Qin, Yan Huang, Hongyun Zhao, Li Zhang

**Affiliations:** Sun Yat-sen University Cancer Center, State Key Laboratory of Oncology in South China, Collaborative Innovation Center for Cancer Medicine, Guangzhou, China; University of North Carolina School of Medicine, United States of America

## Abstract

**Background:**

Multi-targeted antiangiogenic tyrosine kinase inhibitors (MATKIs) have been studied in many randomized controlled trials (RCTs) for treatment of advanced non-small cell lung cancer (NSCLC). We seek to summarize the most up-to-date evidences and perform a timely meta-analysis.

**Methods:**

Electronic databases were searched for eligible studies. We defined the experimental arm as MATKI-containing group and the control arm as MATKI-free group. The extracted data on objective response rates (ORR), disease control rates (DCR), progression-free survival (PFS) and overall survival (OS) were pooled. Subgroup and sensitivity analyses were conducted.

**Results:**

Twenty phase II/III RCTs that involved a total of 10834 participants were included. Overall, MATKI-containing group was associated with significant superior ORR (OR 1.29, 95% CI 1.08 to 1.55, *P* = 0.006) and prolonged PFS (HR 0.83, 0.78 to 0.90, *P* = 0.005) compared to the MATKI-free group. However, no significant improvements in DCR (OR 1.08, 1.00 to 1.17, *P* = 0.054) or OS (HR 0.97, 0.93 to 1.01, P = 0.106) were observed. Subgroup analyses showed that the benefits were predominantly presented in pooled results of studies enrolling previously-treated patients, studies not limiting to enroll non-squamous NSCLC, and studies using MATKIs in combination with the control regimens as experimental therapies.

**Conclusions:**

This up-to-date meta-analysis showed that MATKIs did increase ORR and prolong PFS, with no significant improvement in DCR and OS. The advantages of MATKIs were most prominent in patients who received a MATKI in combination with standard treatments and in patients who had previously experienced chemotherapy. We suggest further discussion as to the inclusion criteria of future studies on MATKIs regarding histology.

## Introduction

Lung cancer is the leading cause of cancer-related mortality worldwide, with about 85% patients diagnosed with non-small cell lung cancer (NSCLC) [1]. Locally advanced or metastatic NSCLC accounts for 80% patients; for these patients the standard care is systemic chemotherapy [2]. Regardless of the emergence of new agents, however, chemotherapy provides only marginal benefit in overall survival [3].

Another treatment option is to inhibit angiogenesis, a complicated process that is regulated by cellular cues, multiple receptor-mediated signaling networks, and a number of pro- and antiangiogenic factors [4], [5]. Antiangiogenic therapy is designed to minimize the acquisition of nutrients and oxygen diffusion to starve tumors. Vascular endothelial growth factor (VEGF) is a key mediator of angiogenesis which has been well studied. Currently, the only antiangiogenic agent approved for patients with NSCLC is bevacizumab, an anti-VEGF monoclonal antibody [6]. However, many other antiangiogenic agents are under clinical development.

VEGF receptor (VEGFR) also plays an important role in the pathways regarding angiogenesis. Multi-targeted antiangiogenic tyrosine kinase inhibitors (MATKIs) are novel agents that target VEGFR-dependent tumor angiogenesis and simultaneously inhibit some other key pathways, such as platelet-derived growth factor (PDGF), fibroblast growth factor (FGF), epidermal growth factor and their associate receptors. Previous studies showed that these small-molecule inhibitors are activitive in a wide variety of cancers [7]. MATKIs could fill in a unique niche for cancer therapeutics, especially in western countries where a relatively small population is suitable for receiving targeted therapies that direct known gene alterations [8]. Recently, these similar MATKIs have showed promising advantages in the treatment of advanced NSCLC [9]. A previous meta-analysis suggested that a regimen of chemotherapy in combination with MATKIs have specific advantages over chemotherapy alone in terms of PFS and ORR, but not in OS [10]. However, it involved only six randomized controlled trials (RCTs) and three agents. Since then, plenty of novel results from phase II/III RCTs have been reported. Thus, we sought to perform a timely meta-analysis to summarize all the evidence including the updated reports. In addition, the abundant data allowed us to carry out some subgroup analyses.

## Methods

### Search Strategy

PubMed, EMBASE, the Cochrane Library as well as the ASCO and ESMO databases from Jan 2005 to Jan 2014 were searched for eligible trials. Search terms were the combination of “non-small-cell lung cancer” with any of the following: “multitargeted antiangiogenesis tyrosine kinase inhibitors” or “sorafenib”, ”sunitinib”, “cediranib”, “vandetanib”, “motesanib”, “nintedanib”, “pazopanib” or “axitinib”. The reference lists of the included studies and recent reviews were checked manually as a supplement. No language restriction was applied.

### Eligibility Criteria

**In order for a study to be included in this analysis,** the following criteria should be met: 1) phase II or III RCT; 2) studies that compared at least one MATKI-containing regimen to MATKI-free regimens as any line treatments in patients with advanced NSCLC; 3) studies reporting at least one response or survival endpoints. In cases of overlap reports, we included only the latest results. Trials will be excluded if they were not in accordance with the eligible criteria.

### Endpoints

The major endpoints for this meta-analysis were overall survival (OS; defined as the time of randomization to the time of death), progression free survival (PFS; defined as the time of randomization to the time of disease progression or death), objective response rate (ORR, percent of patients whose best response was complete response or partial response according to the Recist 1.1 criteria) and disease control rate (DCR, percentage of patients whose best response was complete response, partial response or stable disease according to Recist 1.1 criteria), as well as adverse events (AEs).

### Quality Assessment and Data Extraction

The quality of each eligible study was rated according to the JADAD score [11]. Baseline clinical characteristics, total number of enrolled patients, number of patients who showed complete response, partial response or stable disease, hazard ratio (HR) of median OS and PFS were extracted by two investigators independently. Discrepancies were discussed by the two investigators to reach consensus. In case of missing data, we contacted the primary investigators through emails.

### Statistical Analysis

We defined the experimental arm as MATKI-containing group and the control arm as MATKI-free group. Pooled hazard ratios (HRs) for survival outcomes (PFS and OS) and pooled odds ratio (ORs) for dichotomous data (ORR, DCR and toxicities) with 95% confidence intervals (CI) were calculated using the Inverse Variance algorithm and Mantel-Haenszel algorithm. Heterogeneity across studies was assessed with a forest plot and the inconsistency statistic (I^2^). An I^2^ of 25%, 50% and 75% was considered the cutoff of mild, moderate and severe statistical heterogeneity respectively. A random-effects model was employed in case of the existence of potential heterogeneity; however, both fixed-effect and random effects model were tested. All calculations were performed using STATA 11.0 (StataA Corp, College Station, TX). Subgroup analysis was conducted according to the line of treatment (1^st^-line vs. 2^nd^-/3^rd^-line/maintenance), involved histological type (unselective population vs. selective population for non-squamous carcinoma), the comparison pattern (MATKI + standard treatment/standard treatment vs. MATKI/standard treatment) respectively. Interaction tests were conducted to assess the inter-subgroup differences. Sensitivity analyses were performed. Graphical funnel plots were generated to visually inspect for publication bias. The statistical methods for detecting funnel plot asymmetry were the rank correlation test of Begg and Mazumdar and the regression asymmetry test of Egger [12], [13]. For all analyses, P<0.05 was considered statistically significant.

## Results

### Selection and Features of Included Studies

**Figure 1:**
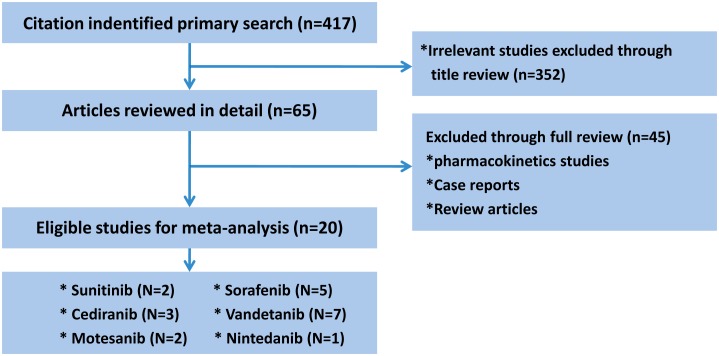
Flow chart of study selection.

Six agents (vandetanib, sunitinib, cediranib, sorafenib, motesanib and nintedanib) could be analyzed while trial data for the other agents remain immature. After screening, 20 phase II/III RCTs (7 for vandetanib, 2 for sunitinib, 3 for cediranib, 5 for sorafenib, 2 for motesanib, and 1 for nintedanib) involving a total of 10834 participants [14]–[33]. Figure 1 showed the flow chart of study selection. The definitions of all studied endpoints were consistent among included studies. Among all studies, 10 trials enrolling patients who previously received chemotherapy while the other 10 included chemo-naïve patients. In addition, five studies limited the eligible histological type to non-squamous carcinoma [18], [20], [31]–[33]. The 2007 study by Heymach et al. [24] contained two dose groups (100 mg/d and 300 mg/d) and the 2008 study by Heymach et al. [27] contained two experimental regimens, they were therefore divided into two separate arms for all analyses. Similarly, Blumenschein’s study [32] was divided into two arms: motesanib 125 mg arm and motesanib 75 mg arm respectively. Table 1 summarized the characteristics of both the included agents and studies.

**Table 1:** Features of included agents and studies.

Agents	Targets	Study byAuthor	Year	Phase	Line	HistologyTypes	Regimens(Experimental arm)	Regimens(Control arm)
**Sunitinib**	VEGFR-1,-2,-3;PDGFRβ; c-kit; Flt-3	Socinski^14^	2010	2	1	All	Bevacizumab+TC+Sunitinib	Bevacizumab+TC
		Scagliotti ^15^	2012	3	2–3	All	Erlotinib+Sunitinib	Erlotinib
**Sorafenib**	b-Raf; VEGFR-2, -3;PDGFRβ; Flt-3; c-kit	Spigel^16^	2011	2	2–3	All	Erlotinib+Sorafenib	Erlotinib
		Scagliotti^17^	2010	3	1	All	TC+Sorafenib	TC
		Paz-Ares^18^	2012	3	1	N-S	GP+Sorafenib	GP
		Wang^19^	2011	2	1	All	GP+Sorafenib	GP
		Molina^20^	2011	2	2	N-S	PEM+Sorafenib	PEM
**Cediranib**	VEGFR-1, -2, -3;PDGFR; FGFR-1; c-kit	Goss^21^	2010	2,3	1	All	TC+Cediranib	TC
		Dy^22^	2013	2	1	All	GC+Cediranib	GC
		Laurie^22^	2012	2,3	1	All	TC+Cediranib	TC
**Vandetanib**	VEGFR-2, -3; EGFR	Heymach^24^	2007	2	2	All	DOC+Vandetanib 100 mg	DOC
						All	DOC+Vandetanib 300 mg	DOC
		Herbst^25^	2010	3	2	All	DOC+Vandetanib 100 mg	DOC
		deBoer^26^	2011	3	2	All	PEM+Vandetanib 100 mg	PEM
		Heymach^27^	2008	2	1	All	TC+Vandetanib 300 mg	TC
						All	Vandetanib 300 mg	TC
		Natale^28^	2011	3	2	All	Vandetanib 300 mg	Erlotinib
		Natale^29^	2009	2	2	All	Vandetanib 300 mg	Gefitinib
		Lee^30^	2012	3	2	All	Vandetanib 300 mg	Placebo
**Motesanib**	VEGFR-1, -2, -3;PDFGR; c-kit	Scagliotti^31^	2012	3	1	N-S	TC+Motesanib	TC
		Blumenschein^32^	2011	2	1	N-S	TC+Motesanib 125 mg	TC+Beva
						N-S	TC+Motesanib 75 mg	TC+Beva
**Nintedanib**	VEGFR 1–3;FGFR 1–3; PDGFR α/β	Martin^33^	2013	3	2	N-S	DOC+Nintedanib	DOC

N-S, non-squamous cell carcinoma; TC, paclitaxel+carboplatin; GP, gemcitabine+cisplatin; DOC, docetaxol; PEM, pemetrxed.

### Meta-analysis of the MATKI-containing Regimens versus MATKI-free Regimens

**Figure 2:**
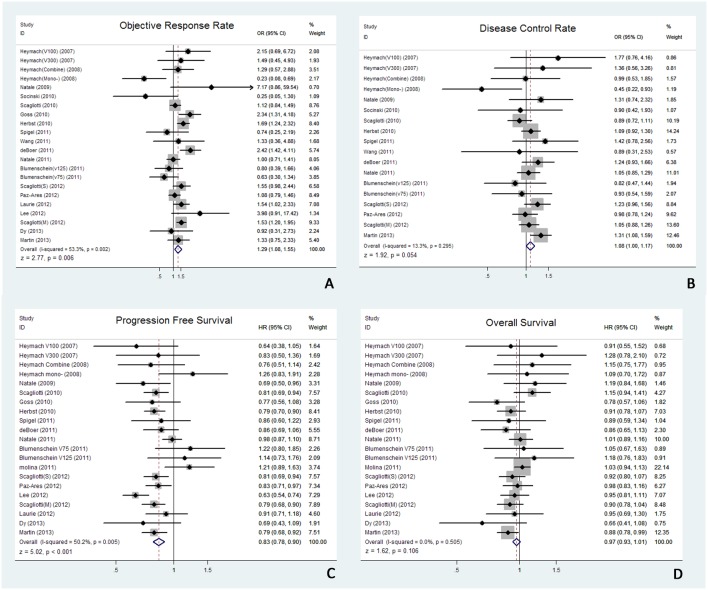
Meta-analyses of MATKIs-containing regimens versus MATKIs-free regimens. A, ORR; B, DCR; C, PFS; D, OS.

When compared to MATKI-free group, MATKI-containing group was associated with significantly superior ORR (OR 1.29, 95% CI 1.08 to 1.55, *P* = 0.006; Fig. 2A) and longer PFS (HR 0.83, 95% CI 0.78 to 0.90, *P* = 0.005; Fig. 2C). However, no significant improvement in DCR (OR 1.08, 95% CI 1.00 to 1.17, *P* = 0.054; Fig. 2B) and OS (HR 0.97, 95% CI 0.93 to 1.01, P = 0.106; Fig. 2D) was observed. Figure 2 illustrates all results of the overall meta-analyses.

### Subgroup Analyses, Sensitivity Analyses and Publication Bias

**Figure 3:**
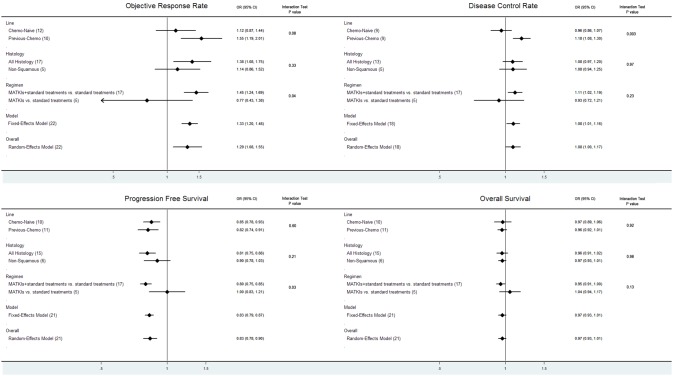
Subgroup analyses of each endpoint (number of studies or arms). A, ORR; B, DCR; C, PFS; D, OS.

When stratifying patients according to chemotherapy history, we observed that greater benefits of MATKIs in ORR and DCR were presented in patients who failed the prior chemotherapy than those without any prior chemotherapy regarding all outcomes (2^nd^-/3^rd^-line/maintenance vs. 1^st^-line: ORR, OR 1.547 vs. 1.116, P_interaction_ = 0.08; DCR, OR 1.183 vs. 0.956, P_interaction_ = 0.003; PFS, HR 0.817 vs. 0.848, P_interaction_ = 0.60; OS, HR 0.965 vs. 0.97, P_interaction_ = 0.92) (greater OR indicated better ORR or DCR, while smaller HR indicated better PFS or OS). In terms of histology, the pooled results of studies that includes tumors of all histological types (not limited to non-squamous NSCLC) showed a favorable trend in comparison with studies selectively enrolled non-squamous carcinoma especially in ORR and PFS (unselective population vs. selective population: ORR, OR 1.375 vs. 1.140, P_interaction_ = 0.33; DCR, OR 1.077 vs. 1.081, P_interaction_ = 0.97; PFS, HR 0.811 vs. 0.899, P_interaction_ = 0.21; OS, HR 0.965 vs. 0.966, P_interaction_ = 0.98). We found that the studies whose experimental arms investigated regimens of adding MATKIs to the regimens in the control arm were associated with greater benefits on PFS (HR 0.798 vs. 0.998, P_interaction_ = 0.03). In addition, significance of DCR improvement (OR 1.095, 95% CI 1.016 to 1.180, *P* = 0.018) and OS benefits (HR 0.951, 95% CI 0.907 to 0.998, P = 0.042) were found to be statistically significant, which differed from the overall results and the results of other subgroups. Conversely, we failed to observe any benefit from studies comparing the efficacy of MATKI to the standard regimens in the control arms. All results of the subgroup analyses (including test of interaction) are illustrated in Figure 3 and were summarized in Table S1.

We conducted a sensitivity analysis by using a fixed-effects model (Table S1). We also conducted a sensitivity analysis to examine the pooled results after excluding one study by lee et al. [30] because it compared vindetanib to placebo which differed significantly from all other included trials. The conclusions regarding all outcomes were not altered in sensitivity analyses. In regard to the publication bias, no significant bias was observed in analyses of all endpoints through both Begg’s test and Egger’s test (P>0.05).

## Discussions

The angiogenesis pathways, which play a critical role in the nourishment of both the tumors and metastatic lesions, have been targeted as a therapeutic option. Bevacizumab, a monoclonal antibody that binds to VEGF, is the only antiangiogenic drug approved for the treatment of NSCLC since it showed overall survival improvement when combined with chemotherapy [6]. An alternative approach is to shut down the VEGFR functions. Therefore, several multi-targeted tyrosine kinase inhibitors (MATKIs) that target VEGFR as well as other key pathways concerning angiogenesis and tumor proliferation are being developed. In some solid tumors, such as renal cell carcinoma and hepatocellular carcinoma, MAKTIs did reduce tumor burden and prolong overall survival [7]. Several of these agents have been evaluated in a series of phase II/III clinical trials for NSCLC patients. A previous meta-analysis suggested that regimens of chemotherapy plus MATKIs were superior to chemotherapy alone in terms of PFS and ORR, but not in OS [10]. However, this meta-analysis involved only six RCTs and three agents (sorafenib, sunitinib, and cediranib). There have been more than ten novel studies published afterwards. Thus, we believed it necessary to update the results using the new evidence and explore some novel information.

According to the current results, regimens containing sorafenib, sunitinib, cediranib, vandetanib, motesanib or nintedanib had substantial improvements for ORR and PFS outcomes, when compared with regimens free of these agents. In contrast, benefits in DCR and OS were not statistically significant. Since ORR is a part of DCR, we speculated that the underlying reason for a difference in associated benefits between ORR and DCR was that MATKIs failed to deliver any additional benefit in patients that presented primary resistance to standard treatments (chemotherapies or TKIs). This hypothesis was strengthened by our findings in the subgroup analyses: MATKIs failed to achieve any improvement when using alone. The current results could be explained by a widely accepted hypothesis that anti-angiogenic agents can transiently normalize the abnormal structure and function of tumor vessels to make it more efficient for drug delivery [34]. Therefore, MATKIs could ‘rescue’ those who actually respond to chemotherapy but suffered from drug accessibility; this finally translated into significantly improved pooled ORR; however patients who were non-responsive remained the same status, therefore there was no significant increase in DCR. Another hypothesis derived from these results was that regulating the VEGFR pathway might play a more important role than inhibition of other targets (EGFR, PDGFR, etc) in the use of MATKIs when no selection for specific patients was conducted. It implied that MATKIs might have to work in combination with other anti-proliferative agents.

With respect to the survival outcomes, the improvement in PFS of MATKIs failed to translate into overall survival benefits. In the perspective of trial design, this might be attributed to the confounding effects from bias of the subsequent treatments. This speculation was supported by the current subgroup analyses which showed that trials studying the maintenance/2^nd^-line/3^rd^-line settings have greater magnitudes of benefits compared with those studying the 1^st^-line settings. This evidence highlighted the advantages of using MATKIs and highlighted the need for balancing post-trial therapies in the future studies.

Since increased risk of hemoptysis in squamous cell carcinoma was documented during the early trials, bevacizumab is only indicated for non-squamous NSCLC [35]. Similarly, part of trials on MATKIs excluded patients with squamous cell carcinoma. Through subgroup analyses, we observed that these trials did not reveal any advantages of MATKI-containing regimens over MATKI-free regimens. However, significant differences were presented when pooling the studies that recruited all histological types (including squamous cell carcinoma). It at least suggested that non-squamous NSCLC might not the targeted subpopulation that benefits most from MATKIs. An exploratory analysis revealed an increased pooled incidence of hemorrhage based on two studies including all histological types, while the incidence was similar between the groups based on the other two studies including only non-squamous NSCLC (data not shown). Although our results did not provide direct evidences, they suggested that patients with squamous cell carcinoma might benefit more from MATKI but might be associated with increased risk for hemorrhage compared with non-squamous NSCLC. However, some recent reports argued that the risk of hemorrhage among squamous NSCLC when using anti-angiogenic agents was acceptable [36]. Therefore, the inclusion criteria, in terms of histology, require further discussion for future studies on MATKIs. Of course, direct comparison of histology associated hemorrhage risk should be proposed to clarify this issue.

This is the most up-to-date comprehensive analysis to compare the MATKI-containing to MATKI-free regimens, confirming the true efficacy of MATKIs. Nevertheless, there are several limitations. Firstly, the meta-analysis might suffer from significant clinical heterogeneity. There were various chemotherapeutic regimens and patterns involved but we were unable to evaluate the respective effect of different MATKIs or treatment settings since current data were not sufficient to draw any solid conclusion for each subset. Secondly, only six agents were finally included in the analysis because studies on other agents were still ongoing (http://clinicaltrials.gov). Thirdly, we were unable to conduct this analysis based on individual patient data, which could definitely provide more reliable information especially in subgroup analyses. To be sure, further studies are warranted to complete the information.

In conclusion, regimens consisting of multi-targeted antiangiogenic TKIs were superior to those without these agents in terms of ORR and PFS in patients with advanced NSCLC. However, no significant benefits in DCR or OS were observed. In addition, subgroup analyses provided us some hints to improve future studies and clinical application of MATKIs.

## Supporting Information

Table S1 **Summary of Subgroup Analyses Results.**
(DOC)

Checklist S1 **PRISMA checklist.**
(PDF)

Flowchart S1 **PRISMA flow chart.**
(PDF)
