# Supplementary material for: Multi-Targeted Antiangiogenic Tyrosine Kinase Inhibitors in Advanced Non-Small Cell Lung Cancer: Meta-Analyses of 20 Randomized Controlled Trials and Subgroup Analyses
Source: PLoS One. 2014 Oct 16;9(10):e109757. doi: 10.1371/journal.pone.0109757 (PMC4199622; doi:10.1371/journal.pone.0109757)
Supplement: Table S1 — Summary of Subgroup Analyses Results. (DOC) [file pone.0109757.s001.doc]

**Table S1. Summary of Subgroup Analyses Results**

| **Outcome** | **Subgroup** | **No. of**  **Studies/Arms** | **OR/HR** | **LL** | **UL** | **Effect Size** | | **Heterogeneity** | |
| --- | --- | --- | --- | --- | --- | --- | --- | --- | --- |
|  |  |  |  |  |  | **Z** | **P-value** | **P-value** | **I^2^** |
| ORR | Chemo-Naive | 12 | 1.116 | 0.866 | 1.438 | 0.85 | 0.396 | 0.004 | 59.70% |
|  | Previous-Chemo | 10 | 1.547 | 1.191 | 2.008 | 3.27 | 0.001 | 0.090 | 40.20% |
|  | All Histology | 17 | 1.375 | 1.081 | 1.751 | 2.59 | 0.01 | 0.003 | 55.50% |
|  | Non-Squamous | 5 | 1.14 | 0.855 | 1.519 | 0.89 | 0.372 | 0.081 | 51.90% |
|  | MATKIs+standard treatments vs. standard treatments | 17 | 1.446 | 1.236 | 1.692 | 4.61 | <0.001 | 0.110 | 30.90% |
|  | MATKIs vs. standard treatments | 5 | 0.77 | 0.429 | 1.382 | 0.88 | 0.381 | 0.029 | 63.00% |
|  | Fixed-Effects Model | 22 | 1.331 | 1.199 | 1.477 | 5.38 | <0.001 | 0.002 | 53.30% |
|  | Random-Effects Model | 22 | 1.294 | 1.078 | 1.552 | 2.77 | 0.006 | 0.002 | 53.30% |
|  |  |  |  |  |  |  |  |  |  |
| DCR | Chemo-Naive | 9 | 0.956 | 0.857 | 1.065 | 0.82 | 0.411 | 0.644 | 0.00% |
|  | Previous-Chemo | 9 | 1.183 | 1.08 | 1.296 | 3.63 | <0.001 | 0.776 | 0.00% |
|  | All Histology | 13 | 1.077 | 0.97 | 1.196 | 1.39 | 0.166 | 0.316 | 12.80% |
|  | Non-Squamous | 5 | 1.081 | 0.938 | 1.247 | 1.08 | 0.281 | 0.218 | 30.60% |
|  | MATKIs+standard treatments vs. standard treatments | 17 | 1.105 | 1.023 | 1.192 | 2.55 | 0.011 | 0.451 | 0.00% |
|  | MATKIs vs. standard treatments | 5 | 0.934 | 0.719 | 1.213 | 0.51 | 0.61 | 0.184 | 35.60% |
|  | Fixed-Effects Model | 18 | 1.084 | 1.011 | 1.162 | 2.26 | 0.024 | 0.295 | 13.30% |
|  | Random-Effects Model | 18 | 1.081 | 0.999 | 1.171 | 1.92 | 0.054 | 0.295 | 13.30% |
|  |  |  |  |  |  |  |  |  |  |
| PFS | Chemo-Naive | 10 | 0.848 | 0.777 | 0.926 | 3.68 | <0.001 | 0.266 | 19.20% |
|  | Previous-Chemo | 11 | 0.817 | 0.735 | 0.909 | 3.72 | <0.001 | 0.001 | 65.40% |
|  | All Histology | 15 | 0.811 | 0.745 | 0.884 | 4.8 | <0.001 | 0.015 | 49.70% |
|  | Non-Squamous | 6 | 0.899 | 0.783 | 1.033 | 1.5 | 0.133 | 0.037 | 57.80% |
|  | MATKIs+standard treatments vs. standard treatments | 17 | 0.798 | 0.752 | 0.846 | 7.5 | <0.001 | 0.205 | 21.80% |
|  | MATKIs vs. standard treatments | 5 | 0.998 | 0.826 | 1.205 | 0.02 | 0.985 | 0.119 | 45.50% |
|  | Fixed-Effects Model | 21 | 0.828 | 0.792 | 0.865 | 8.38 | <0.001 | 0.005 | 50.20% |
|  | Random-Effects Model | 21 | 0.835 | 0.778 | 0.896 | 5.02 | <0.001 | 0.005 | 50.20% |
|  |  |  |  |  |  |  |  |  |  |
| OS | Chemo-Naive | 10 | 0.97 | 0.888 | 1.06 | 0.67 | 0.502 | 0.363 | 8.70% |
|  | Previous-Chemo | 11 | 0.965 | 0.919 | 1.014 | 1.41 | 0.158 | 0.494 | 0.00% |
|  | All Histology | 15 | 0.965 | 0.909 | 1.025 | 1.17 | 0.243 | 0.534 | 0.00% |
|  | Non-Squamous | 6 | 0.966 | 0.926 | 1.007 | 1.04 | 0.299 | 0.273 | 21.30% |
|  | MATKIs+standard treatments vs. standard treatments | 17 | 0.951 | 0.907 | 0.998 | 2.03 | 0.042 | 0.388 | 5.70% |
|  | MATKIs vs. standard treatments | 5 | 1.045 | 0.935 | 1.168 | 0.77 | 0.439 | 0.891 | 0.00% |
|  | Fixed-Effects Model | 21 | 0.966 | 0.926 | 1.007 | 1.62 | 0.106 | 0.505 | 0.00% |
|  | Random-Effects Model | 21 | 0.966 | 0.926 | 1.007 | 1.62 | 0.106 | 0.505 | 0.00% |
