# Supplementary material for: Multi-Targeted Antiangiogenic Tyrosine Kinase Inhibitors in Advanced Non-Small Cell Lung Cancer: Meta-Analyses of 20 Randomized Controlled Trials and Subgroup Analyses
Source: PLoS One. 2014 Oct 16;9(10):e109757. doi: 10.1371/journal.pone.0109757 (PMC4199622; doi:10.1371/journal.pone.0109757)
Supplement: Flowchart S1 — PRISMA flow chart. (PDF) [file pone.0109757.s003.pdf]

PRISMA 2009 Flow Diagram

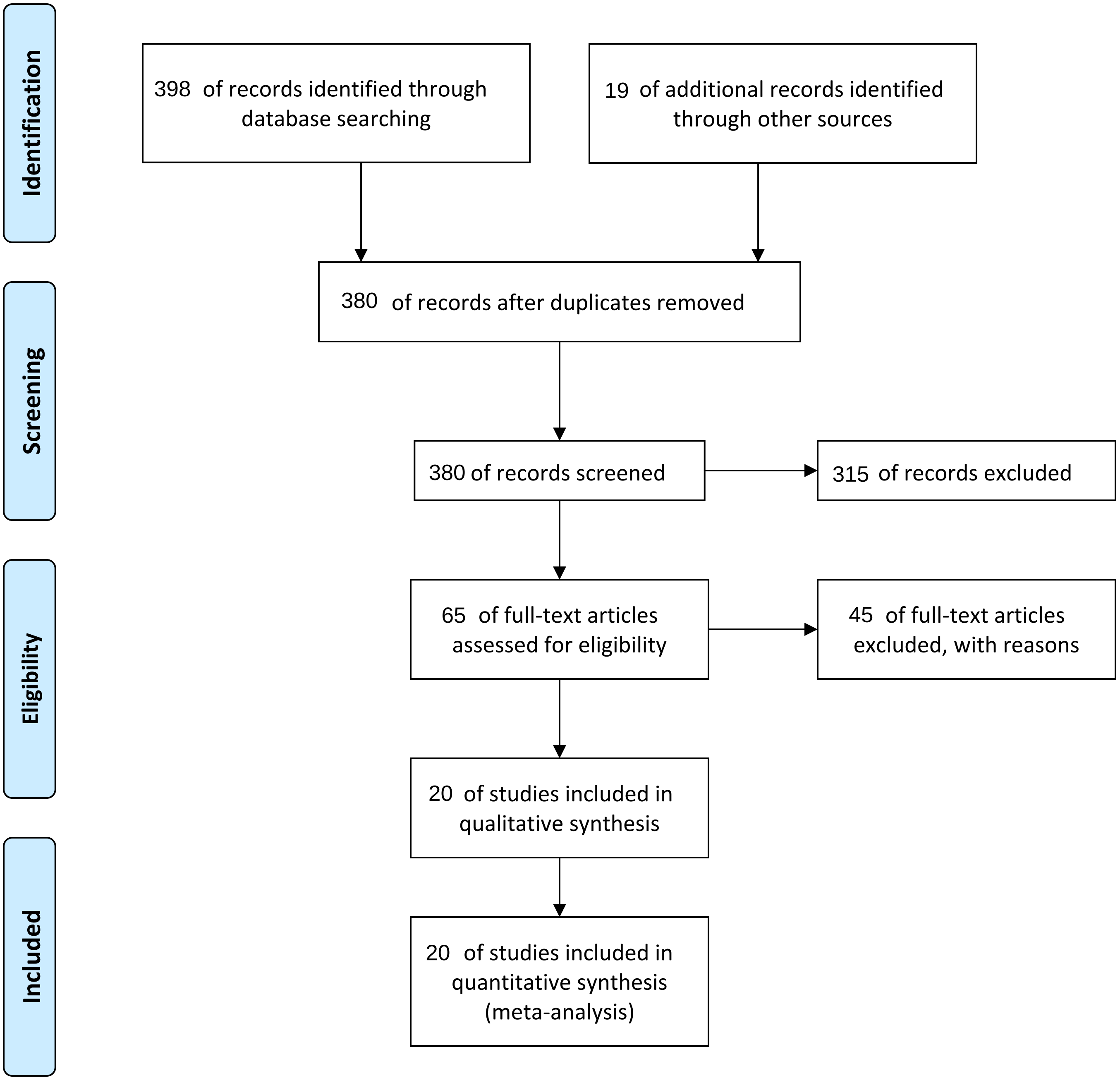

From: Moher D, Liberati A, Tetzlaff J, Altman DG, The PRISMA Group (2009). *P*referred *R*eporting *I*tems for *S*ystematic Reviews and *M*eta-*A*nalyses: The PRISMA Statement. PLoS Med 6(6): e1000097. doi:10.1371/journal.pmed1000097

For more information, visit www.prisma-statement.org.
